# Unexpected Neurological Deterioration Following Occipitocervical Fusion Surgery: A Case Report

**DOI:** 10.7759/cureus.78129

**Published:** 2025-01-28

**Authors:** Takane Nakagawa, Hiroshi Takahashi, Kousei Miura, Toru Funayama, Masao Koda

**Affiliations:** 1 Department of Orthopaedic Surgery, Institute of Medicine, University of Tsukuba, Tsukuba, JPN

**Keywords:** atlantoaxial subluxation, high cervical myelopathy, instrumentation failure, occipitocervial fusion surgery, os odontoideum, triple rod

## Abstract

Occipitocervical posterior decompression and fusion (O-C fusion) surgery is occasionally required for treating high cervical myelopathy due to atlantoaxial subluxation. The advance of the instrumentation systems has led to favorable clinical outcomes following O-C fusion surgery. However, the rate of perioperative complications in O-C fusion surgery is relatively high, including instrumentation failure, respiratory complications, and dysphagia. Here we report a rare case involving an unexpected deterioration of myelopathy following O-C fusion surgery. A 49-year-old male was transported to our hospital by ambulance with left-sided upper and lower limb paralysis. At the initial visit, a neurological examination revealed left upper limb weakness (manual muscle testing (MMT) grade 2). X-ray and CT revealed severe atlantoaxial subluxation due to os odontoideum, while MRI revealed significant spinal cord compression at the C2 level. On the diagnosis of acute exacerbation of high cervical myelopathy, an O-C2 posterior decompression and fusion surgery, including C1 laminectomy, was performed. Postoperatively, the patient exhibited a deterioration in right-sided upper limb paralysis (MMT grade 2), despite proper implant placement confirmed by CT. During two weeks postoperatively, there was no improvement in the right-sided upper limb paralysis, and bilateral deep sensory impairment worsened. Follow-up X-rays revealed a progressive decrease in the O-C2 angle, and dynamic X-ray imaging demonstrated a recurrence of instability at the O-C2 level. On the diagnosis of the instrumentation failure, a revision surgery was performed three weeks after the primary surgery. Intraoperative findings revealed instability at the C2 screw head and loosening of the set screw on the C2 screw head. To achieve a more secure fixation, we extended the fusion to C4 with a triple rod connection. Following the revision surgery, his myelopathy and paralysis gradually improved. At the final follow-up, six months after surgery, X-rays showed that O-C2 was firmly stabilized. In conclusion, screw head fixation close to the O-C rod bending site may result in unexpected instrumentation failure in O-C fusion surgery.

## Introduction

Occipitocervical posterior decompression and fusion (O-C fusion) surgery is occasionally required to treat high cervical myelopathy associated with atlantoaxial subluxation. Recent advancements in instrumentation techniques have contributed to improved clinical outcomes [1,2]. Nevertheless, the rate of perioperative complications in this procedure is relatively high, including instrumentation failure, respiratory complications, and dysphagia [3-7]. Here we report a rare case involving an unexpected deterioration of myelopathy following O-C fusion surgery.

This article was previously posted to the Research Square preprint server on November 18, 2024.

## Case presentation

**Figure 1:**
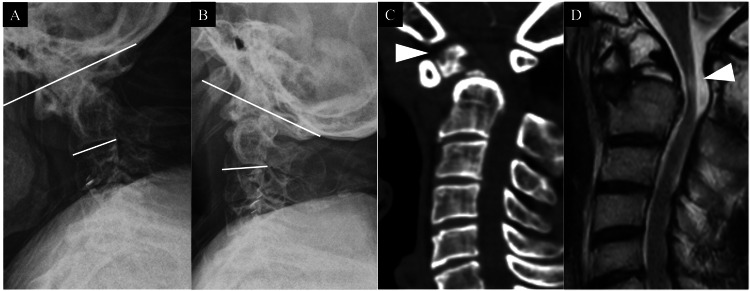
Initial cervical imaging. A: at flexion (O-C2 angle: -11.3°); B: at extension (O-C2 angle: 33.8°); C: CT shows os odontoideum (arrowhead); D: MRI (T2WI) shows spinal cord compression and T2 high-signal change at C2 level (arrowhead). T2WI: T2-weighted image

In January 2023, a 49-year-old Japanese man was transported to our hospital by ambulance after experiencing a loss of consciousness and weakness in his left upper and lower limbs while working. At the initial examination, manual muscle testing (MMT) revealed grade 2 muscle weakness in the distal muscles of the left upper limb, while the lower limbs exhibited no detectable muscle weakness. Superficial sensation remained intact, and deep tendon reflexes were hyperactive in all four extremities. The Japanese Orthopaedic Association (JOA) score for cervical myelopathy was six points out of 17. The X-ray revealed significant instability, with an O-C2 angle measuring -11.3° at flexion and 33.8° at extension (Figure 1A, 1B). Computed tomography (CT) revealed the presence of os odontoideum and atlantoaxial subluxation (Figure 1C). Magnetic resonance imaging (MRI) demonstrated anterior spinal cord compression and intramedullary T2 high-signal change at the C2 level (Figure 1D).

**Figure 2:**
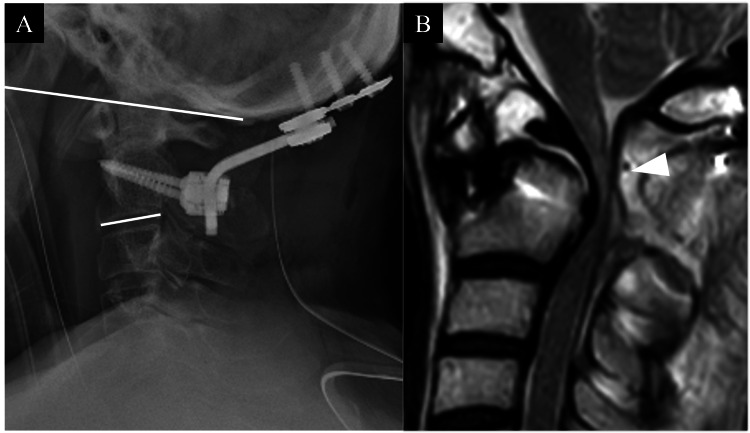
Postoperative cervical imaging. A: postoperative X-ray (O-C2 angle was 15.7°); B: after initial surgery, MRI (T2WI) shows remaining and exacerbation of cord compression (arrowhead). T2WI: T2-weighted image

On the diagnosis of acute exacerbation of high cervical myelopathy due to atlantoaxial subluxation associated with os odontoideum, O-C fusion surgery was performed (Figure 2A). The surgery was involved in the removal of the C1 posterior arch, insertion of bilateral C2 pedicle screws, placement of an occipital plate, manual reduction of the C1/2 subluxation, fixation with 3.5 mm diameter rods, and iliac bone grafting.

Postoperatively, the patient was managed with intubation in the High Care Unit based on the anesthesiologist’s decision and was extubated the following day. Neurological evaluation conducted the day after surgery revealed progression of paralysis in bilateral upper limbs (MMT: left upper limb grade 3, right upper limb grade 2) and deep sensory disturbances in both lower extremities. The JOA score for cervical myelopathy deteriorated to 3.5 points out of 17. Postoperative CT revealed no issues with screw positioning. Despite additional support with a Philadelphia neck collar immobilization, there was no improvement in paralysis. One week after the surgery, muscle weakness in the right lower limb and bilateral deep sensory disturbances worsened. Postoperative MRI revealed persistent anterior spinal cord compression at the C2 level despite intraoperative reduction of C1/2 (Figure 2B).

**Figure 3:**
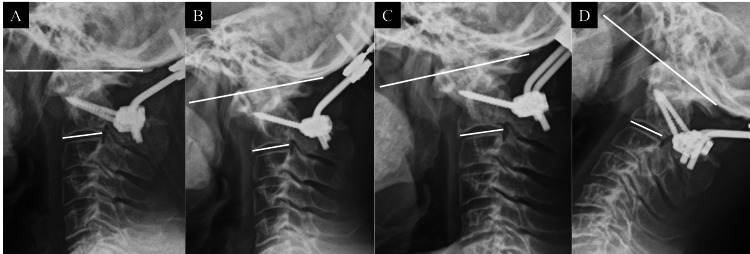
Following up X-ray. A: postoperative one week at neutral position (O-C2 angle: 8.3°); B: postoperative two weeks at neutral position (O-C2 angle: 2.2°), O-C2 angle decreased gradually; C: postoperative three weeks at flexion (O-C2 angle:-1.3°); D: postoperative three weeks at extension (O-C2 angle:-8.2°), functional radiograph shows instability at O-C2 level.

Upon serial examination of postoperative X-rays, a gradual loss of correction was observed in the O-C2 angle over time. The intermediate lateral view demonstrated an O-C2 angle of 15.7° immediately post-surgery, which decreased to 8.3° at one week (Figure 3A) and further reduced to 2.2° at two weeks postoperatively (Figure 3B). Furthermore, dynamic X-ray imaging at three weeks postoperatively demonstrated an O-C2 angle of -1.3° at flexion and 8.2° at extension. Notably, these images revealed evident instability of the C2 pedicle screw heads during flexion extension, suggesting fixation failure (Figure 3C, 3D).

**Figure 4:**
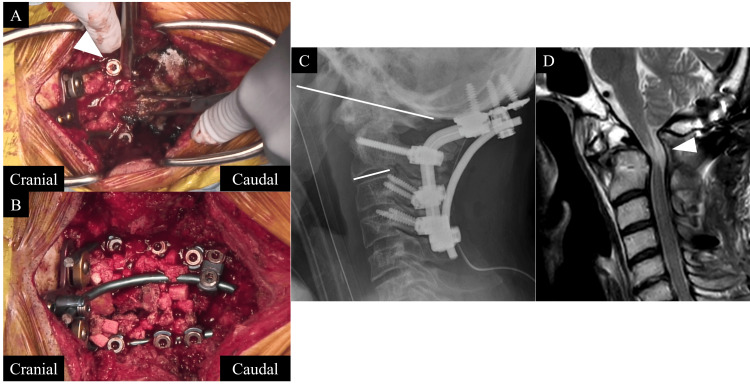
Intraoperative findings and postoperative imaging after revision surgery. A: intraoperative findings at revision surgery revealed instability at the C2 screw head and loosening of the set screw on the C2 screw head (arrowhead); B: revision surgery, extending fusion to C4 and adding the triple rod; C: O-C2 angle was improved to 25°; D: after revision surgery, MRI (T2WI) shows improving cord compression (arrowhead). T2WI: T2-weighted image

Given these findings, a revision surgery was performed three weeks following the initial surgery. Intraoperative findings revealed that the C2 screw heads were unstable due to their attachment at the rod bending points, and the set screws were loosened (Figure 4A). All implants were removed and replaced with a 4.0 mm rod system; additional lateral mass screws were placed in C3 and C4, and the O-C2 angle was adjusted and secured with additional rods for reinforcement (Figure 4B).

**Figure 5:**
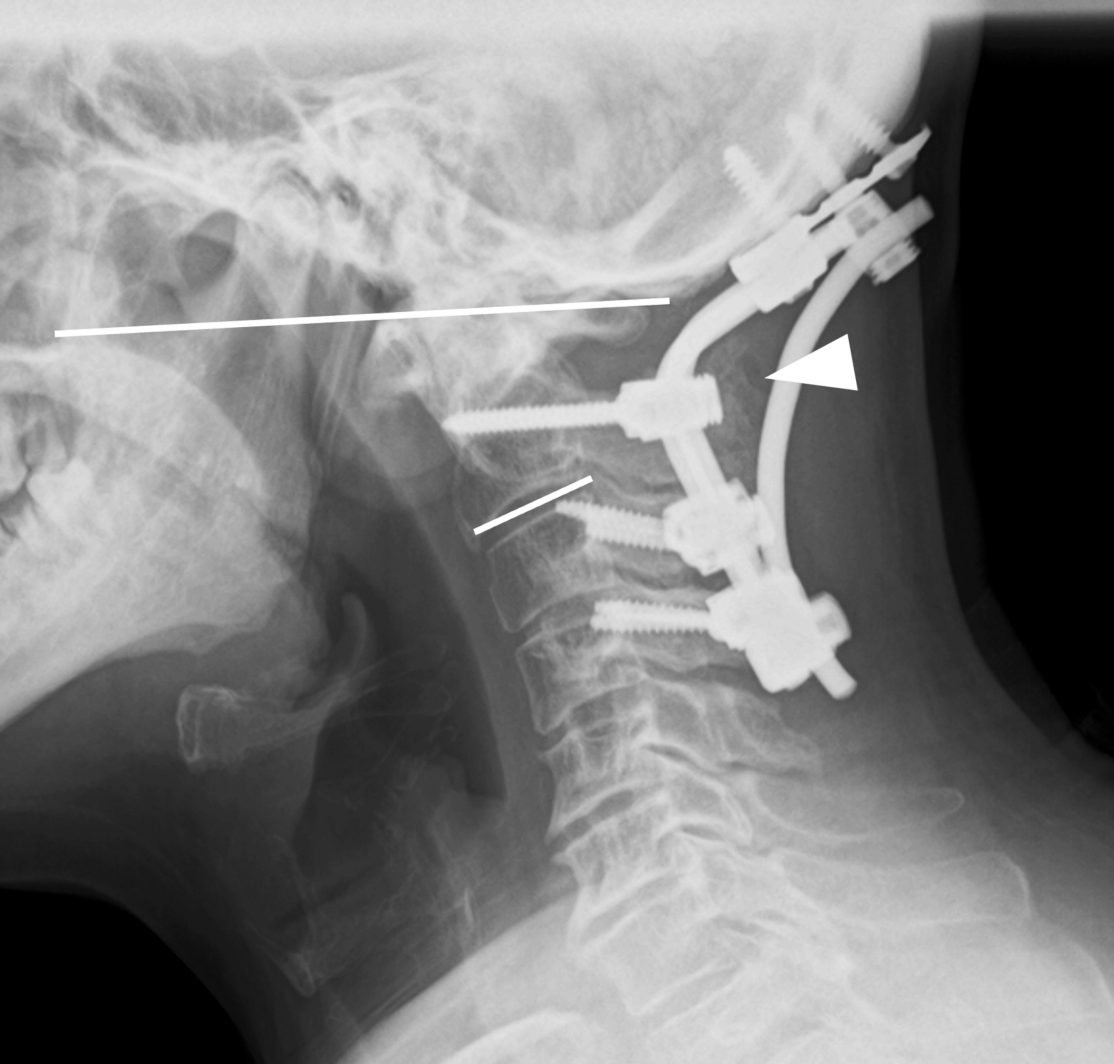
X-ray lateral image one year after surgery. The O-C2 angle was maintained without loss of correction. In addition, the posterior bony fusion was observed (arrowhead).

After the revision surgery, his paralysis of lower extremities gradually improved (MMT: right upper limb 3, left upper limb 4 at two weeks). Furthermore, his deep sensory disturbances also improved, allowing the patient to start gait training. Postoperative X-ray demonstrated adequate reduction with an O-C2 angle of 25 degrees (Figure 4C). MRI revealed an expansion of the posterior subarachnoid space, indicating successful posterior decompression of the spinal cord (Figure 4D). At the final follow-up, one year after surgery, the JOA score for cervical myelopathy improved to 10 points out of 17. The O-C2 angle was maintained without loss of correction, indicating posterior fusion (Figure 5).

## Discussion

**Figure 6:**
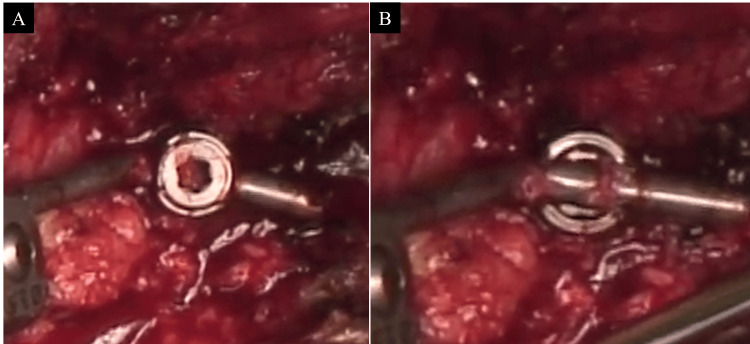
Intraoperative findings of revision surgery (magnified). A: the intraoperative findings revealed that the set screw was loosening; B: after the set screw was removed. The C2 screw head was positioned close to the rod bending site.

Instrumentation failure in high cervical instrumented surgery, such as rod fracture and screw loosening or screw breakage, has been occasionally observed in the literature [4,8-10]. However, to the best of our knowledge, there have been no reports of instrumentation failure due to loosening of the set screws at the screw heads, as encountered in the present case. During the initial surgery, the set screws were tightened to the appropriate torque, and sufficient fixation was achieved intraoperatively. Nevertheless, in the present case, set screw loosening occurred in the early postoperative phase. We hypothesized that this phenomenon was caused by the attachment of the C2 set screws at the rod bending sites. In O-C fusion surgery, it is reported that an inappropriately small O-C2 angle can lead to dysphagia and respiratory complications [3]. However, attempting to increase the O-C2 angle inevitably brings the C2 screw heads closer to the rod bending sites. In addition, steep rod bending is often required to increase the O-C2 angle. Consequently, the set screws may not be able to adequately secure the rod at the screw head, leading to insufficient fixation. Therefore, in cases requiring a large O-C2 angle, it is important to be aware of the potential for the C2 screw heads to be positioned close to the rod bending sites, as observed in the present case (Figure 6A, 6B).

During the revision surgery for the present case, we extended the fixation to C4 and reinforced the instrumentation with a triple rod system to achieve more reliable and robust fixation. In O-C fusion surgery, there are some reports discussing methods to reinforce the instrumentation to prevent early instrumentation failure [9,11]. In retrospect, for cases with severe instability like the present case, it might have been better to increase the number of fixation anchors during the initial surgery.

## Conclusions

In O-C fusion surgery, particularly in cases requiring a large O-C2 angle, it is crucial to be aware that set screw fixation at the rod bending sites can lead to unexpected early instrumentation failure. Careful consideration of rod application is essential to prevent this phenomenon.
